# Difficult diagnosis of tetanus in a sedated patient after ileal resection

**DOI:** 10.1186/s40981-025-00779-8

**Published:** 2025-03-08

**Authors:** Satoko Noguchi, Junichi Saito, Eiji Hashiba

**Affiliations:** Department of Anesthesiology, Hirosaki University Graduate School of Medicine, 5 Zaifu-Cho, Hirosaki, Aomori 036-8562 Japan

**Keywords:** Tetanus, Bacterial translocation, Muscle rigidity

## Abstract

**Background:**

We report a case of tetanus developed after inguinal hernia repair without traumatic wounds, which was difficult to be diagnosed under sedation with mechanical ventilation for pneumonia and anaphylactic shock.

**Case presentation:**

The 67-year-old Japanese male underwent inguinal hernia repair with ileal resection. On postoperative day (POD) 9, he was admitted to the ICU due to dyspnea and worsening oxygenation. Immediately after tazobactam piperacillin was administered as empiric treatment for aspiration pneumonia, anaphylaxis developed, requiring tracheal intubation and continuous intravenous adrenaline. On POD 10, sedative titration increased his extremities’ muscle rigidity; weaning from mechanical ventilation was difficult, while he could communicate. On POD 15, tetanus was diagnosed based on the physical examination findings prior to ICU admission.

**Conclusion:**

Tetanus should be considered when patients show abnormal hypertension and no impaired consciousness during muscle rigidity, even in the absence of obvious trauma.

## Background

Tetanus is a neurological infectious disease caused by *Clostridium tetani* (an anaerobic G-negative bacillus), which produces tetanospasmin [1]. Tetanospasmin acts on the presynapses of inhibitory neurons and inhibits the release of neurotransmitters, resulting in tetanus-specific neurologic symptoms such as lockjaw and posterior neck stiffness. Although traumatic wounds usually trigger tetanus with a 3- to 5-day incubation period, several cases of tetanus with minor trauma or no prior trauma have been reported [2–5]. We report a case of tetanus that was identified after an ileal resection without trauma. It was difficult to diagnose the tetanus because the patient was being treated for pneumonia and anaphylactic shock.

## Case presentation

A 67-year-old Japanese male apple farmer (height 157 cm, weight 40 kg), who had no remarkable medical history, was admitted to our hospital’s emergency department with difficulty moving and a right inguinal swelling that had not improved over the past few days. A computed tomography (CT) scan showed a right inguinal incarcerated hernia without blood flow obstruction. Since a manual repair of the hernia was unsuccessful, an inguinal hernia repair with ileal resection was performed.

After the ileal resection surgery, the patient was admitted to the intensive care unit (ICU) for acute kidney injury and tachycardic atrial fibrillation due to dehydration. The acute kidney injury and tachycardia were improved after a blood transfusion, and the patient was discharged to the general ward on postoperative day (POD) 1. Prolonged ileus was managed with fluid replacement and decompression with an ileus tube. As psychological disturbance had appeared, delirium was suspected at POD 8. Mild spasm-like movements were observed in the patient’s extremities, but no disturbance of consciousness or paralysis was observed.

On the night of POD 9, the patient was readmitted to the ICU due to difficulty in excreting sputum and a deterioration of oxygenation. Respiratory condition improved after bronchoscopy and hemodynamics was stable, but we observed muscle rigidity throughout his body at the slightest contact or noise, such as drawing a curtain or calling his name. Blood gas analysis at the admission to the ICU was almost normal (pH 7.32, PaO_2_ 139 Torr, PaCO_2_ 45 Torr, base excess − 2 mmol/L under 6 L/min of oxygen mask administration), and the sinus tachycardia in the HR 110–120 beats/min. Just after an administration of tazobactam piperacillin as empirical therapy for aspiration pneumonia, his systolic blood pressure suddenly dropped to 40 mmHg, transcutaneous oxygen saturation decreased to 50%, and a rapid skin rash appeared over the entire body, which was diagnosed as anaphylactic shock. Blood gas analysis showed a marked acidosis (pH 7.07, PaO_2_ 48 Torr, PaCO_2_ 86 Torr, base excess − 8 mmol/L under mask ventilation), requiring tracheal intubation and continuous intravenous adrenaline administration with a maximum dose of 0.15 μg/kg/min.

The following day (POD 10), the adrenaline was discontinued but the muscle rigidity in the patient’s extremities increased with the tapering of propofol (2–3 mg/kg/h) and dexmedetomidine (0.4 μg/kg/h), making a withdrawal of the mechanical ventilation difficult. Blood cultures, a cerebrospinal fluid (CSF) analysis, and electroencephalography (EEG) were negative for meningitis and epilepsy. However, the patient’s irritability gradually worsened, and his tidal volume temporally decreased from 8 to 3 mL/kg due to muscle rigidity. Respiratory rate was stable at about 10 breaths/min under sedation but tachypnea of more than 25 breaths/min was noted with awakening. Abnormal hypertension and severe blood pressure fluctuations were also observed, and systolic blood pressure temporarily rose to 180 mmHg on awakening while requiring continuous intravenous noradrenaline 0.1 μg/kg/min during sedation. The patient was nevertheless able to communicate even during periods of muscle rigidity, and he complained of severe pain throughout his body due to muscle rigidity. This required frequent propofol flushes for sedation.

During our consideration of the correct diagnosis and appropriate treatment, a review of the patient’s course and history revealed that he showed difficulty in opening the mouth during bronchoscopy in the general ward, which is a key physical finding in the diagnosis of tetanus. Based on this information, tetanus was finally diagnosed on POD 15; metronidazole and human tetanus globulin were administered the same day, and a continuous intravenous administration of rocuronium (7 μg/kg/min) was started.

A tracheostomy was performed on POD 16. Although deep sedation of the patient was required for a few more days, his ileus tended to improve and tube-feeding was started. The fluctuation of the patient’s blood pressure also improved. On POD 25, intravenous rocuronium was discontinued, and continuous propofol was replaced with continuous midazolam, as muscle rigidity was more likely to flare. The patient was discharged from the ICU on POD 30 and weaned from the ventilator on POD 36. His ileus worsened again but improved on POD 58, and he was transferred to a rehabilitation facility on POD 61.

The patient’s fully informed written consent to publish this case report was obtained.

## Discussion

The clinically important issues highlighted by this patient’s case are that (i) the diagnosis of tetanus in a sedated patient can be difficult, and (ii) tetanus can be caused by a bacterial translocation other than that involving trauma. Although trauma is the most common cause of tetanus, 4–30% of cases are reported to have no clear history of trauma [6–8]. Cases of tetanus have been described in individuals with frequent daily contact with soil (e.g., civil engineers and agriculture workers) and individuals with burns, surgical wounds, delivery wounds, or hemorrhoids and after tooth extraction [1]. Although the present case of tetanus was not detected by a wound culture, the patient was an agricultural worker and may have developed tetanus as a postoperative complication that included bacterial translocation due to the incubation period and prolonged ileus.

Kato et al. detected tetanus bacilli in drain cultures after ileus surgery, and they noted that the therapeutic intervention was performed before the appearance of clinical symptoms [9]. The case of an elderly woman who developed tetanus after ileus surgery was described by Mori et al. [10], and Imai et al. reported a similar case of an elderly woman who developed tetanus on the fifth postoperative day [11]; however, in both patients the results of all culture tests including blood cultures were negative. No tetanus culture was detected in our present patient, and it was difficult to identify his tetanus based on information other than the clinical findings.

An additional clinically important issue highlighted by our patient’s case is that the absence of impaired consciousness during periods of muscle rigidity and abnormal hypertension are both helpful in the diagnosis of tetanus. Severe blood pressure fluctuation is an important symptom of tetanus caused by sympathetic hypertonia. In addition, the ability to communicate during muscle rigidity is a major difference from central nervous system disease and should be emphasized as a point of differentiation.

In the case reports cited above, tetanus could be suspected even in the absence of obvious trauma due to the appearance of dysphagia and muscle rigidity. In contrast, our patient was only slightly symptomatic but required tracheal intubation under sedation for an entirely different reason, making the diagnosis of tetanus very difficult. The diagnosis took 5 days from the onset to the diagnosis, and interventions such as an administration of muscle relaxants and management in a dark room were delayed. This case suggests that it is important to consider tetanus as a differential diagnosis in cases of muscle stiffness that has arisen for an unknown reason, and to consider the patient’s living/work environment and possible wound infection [2, 8].

In conclusion, the diagnosis of tetanus in a sedated patient can be difficult, but it is important to keep in mind that tetanus can also be caused by bacterial translocation other than that involving trauma and should be included in differential diagnoses in similar cases. The presence of abnormal hypertension and the absence of impaired consciousness during muscle rigidity are helpful in the diagnosis of tetanus.

## Data Availability

The corresponding author can be contacted for data requests.

## Abbreviations

CSF: Cerebrospinal fluid
EEG: Electroencephalography
ICU: Intensive care unit
POD: Postoperative day
